# Moving Protein PEGylation from an Art to a Data Science

**DOI:** 10.1021/acs.bioconjchem.2c00262

**Published:** 2022-08-22

**Authors:** Leran Mao, Alan J. Russell, Sheiliza Carmali

**Affiliations:** † Department of Chemical Engineering, Carnegie Mellon University, Pittsburgh, Pennsylvania 15213, United States; ‡ Amgen Inc., Thousand Oaks, California 91320, United States; § School of Pharmacy, Queen’s University Belfast, Belfast, BT9 7BL United Kingdom

## Abstract

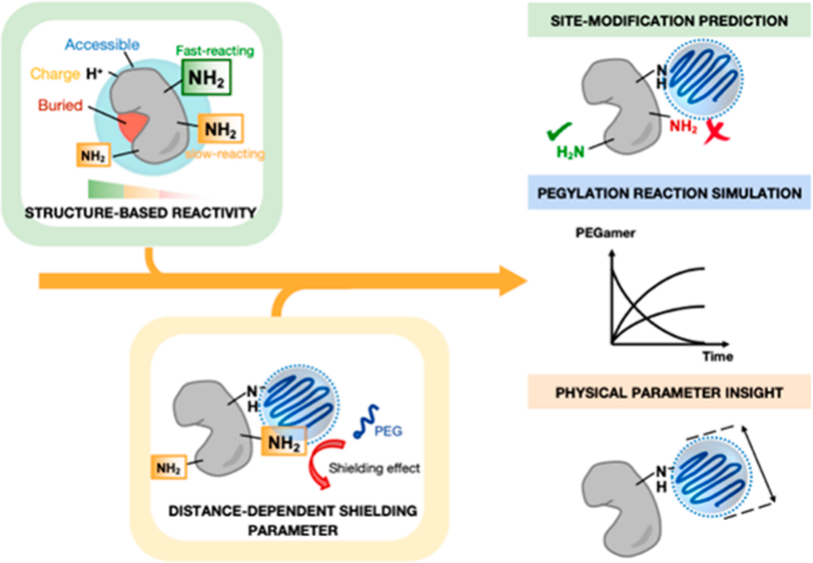

PEGylation is a well-established and clinically proven half-life extension strategy for protein delivery. Protein modification with amine-reactive poly(ethylene glycol) (PEG) generates heterogeneous and complex bioconjugate mixtures, often composed of several PEG positional isomers with varied therapeutic efficacy. Laborious and costly experiments for reaction optimization and purification are needed to generate a therapeutically useful PEG conjugate. Kinetic models which accurately predict the outcome of so-called “random” PEGylation reactions provide an opportunity to bypass extensive wet lab experimentation and streamline the bioconjugation process. In this study, we propose a protein tertiary structure-dependent reactivity model that describes the rate of protein-amine PEGylation and introduces “PEG chain coverage” as a tangible metric to assess the shielding effect of PEG chains. This structure-dependent reactivity model was implemented into three models (linear, structure-based, and machine-learned) to gain insight into how protein-specific molecular descriptors (exposed surface areas, p*K*_a_, and surface charge) impacted amine reactivity at each site. Linear and machine-learned models demonstrated over 75% prediction accuracy with butylcholinesterase. Model validation with Somavert, PEGASYS, and phenylalanine ammonia lyase showed good correlation between predicted and experimentally determined degrees of modification. Our structure-dependent reactivity model was also able to simulate PEGylation progress curves and estimate “PEGmer” distribution with accurate predictions across different proteins, PEG linker chemistry, and PEG molecular weights. Moreover, in-depth analysis of these simulated reaction curves highlighted possible PEG conformational transitions (from *dumbbell* to *brush*) on the surface of lysozyme, as a function of PEG molecular weight.

## Introduction

PEGylated protein conjugates are widely used as therapeutics. Since the approval of Adagen and Oncaspar in the 1990s, more than 20 PEGylated drugs have been approved by the Food and Drug Administration, with many more in clinical development.^1,2^ Over the years, the prominent success of PEGylation has led to innovative conjugation chemistries for improved site-specificity and reduced impact on protein function. Despite this, most approved PEGylated drugs are still obtained by “random” nonspecific lysine-targeted chemistry. This “randomness” results in heterogeneous mixtures of native protein and “PEGmers” (i.e., mono-, di-, tri-PEGylated protein species) which require extensive reaction optimization and purification. We place the word random in quotation marks because, even though lysine modification has been described as “random” for decades, the reaction outcome is not random at all and should be predictable from first principles. Individual PEG-protein species can also differ in modification site, with each positional PEG isomer having differential effects on pharmacologic, toxicologic, and immunogenic activity. For example, PEG-interferon alpha-2a (PEGASYS) is composed of nine positional isomers, each displaying significant differences in efficacy.^3^ Researchers therefore need to perform time-consuming and costly stochastic experiments to generate PEGylated conjugate mixtures with a desired average therapeutic profile. The ability to optimize reaction conditions and predict where, and to what extent, lysine residues can be modified with amine-reactive PEGs would greatly improve the efficiency of bioconjugation.

Previously, our group has shown that the tertiary structure of a protein can be used to assess the relative reactivity at each lysine residue.^4^ We developed a decision tree based on experimental data which was used to predict the relative reaction between amino groups in a protein and an *N*-hydroxysuccinimide small molecule compound. To automate the process and create a widely accessible bioconjugation tool, we devised PRELYM,^5^ a Python program that implements the decision tree to provide qualitative insight into which lysines in a given protein are most likely to react and their relative order of reaction.

Kinetic models,^6−12^ which provide broader insights to reaction mechanisms, laboratory-, and industrial-scale process development, have the potential to help automate the bioconjugation process through rapid online parameter optimization in a design of experiment (DOE)-like manner.^13−15^ For lysine reactions, the multiplicity of conjugation sites has made kinetic modeling challenging. Early PEGylation kinetic models were able to simulate the degree of PEGylation but not distinguish positional PEG isomers. Moreover, those models frequently used lumped parameters to empirically define the rate of reaction, thereby limiting the possibility to extract physical parameters and reducing generalizability. A more recent and improved model, developed by Pfister and co-workers, describes amine PEGylation by considering the intrinsic reactivity of primary amines, steric hindrance from PEG shielding (once PEG has reacted at one site, further reactions at near neighbor sites will be sharply diminished), and diffusional constraints that slow the reaction.^12^ However, in this impressive iso-kinetic reactivity model, shielding is defined in a way that the kinetic rate is only affected by the extent of PEGylation and not by the site of PEGylation. Indeed, Pfister’s iso-reactivity model assumes an equal intrinsic reactivity of each site. While this is valid for a protein with a low degree of PEGylation, lower intrinsic reactivities of subsequent reaction sites can be incorrectly predicted as reduced reactivity through shielding, resulting in inaccurate interpretations of physical parameters.

**Figure 1:**
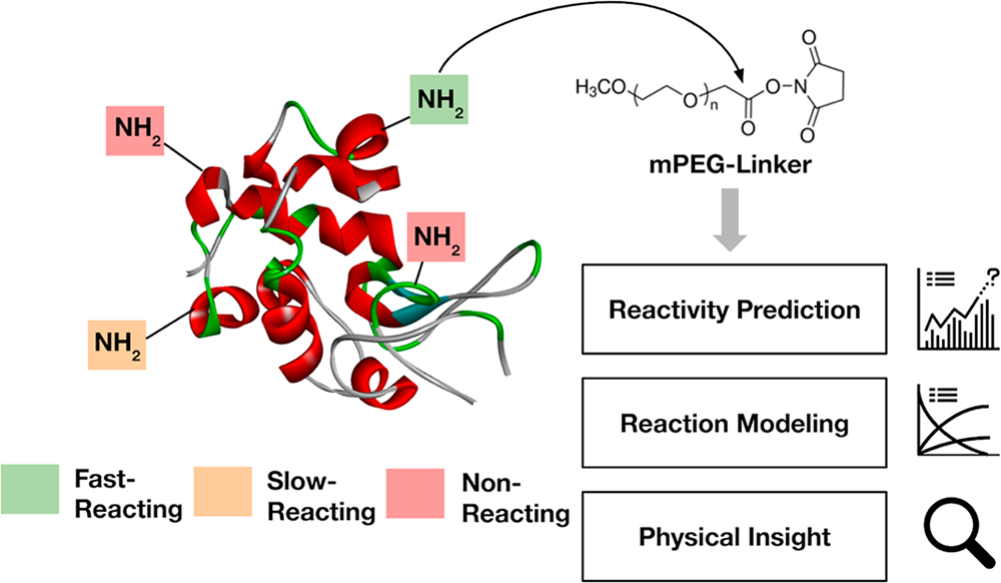
Overview of the structure-dependent reactivity kinetic model which can provide site-modification predictions and simulate PEGylation reactions.

For decades, the field of protein PEGylation has needed a comprehensive model that would identify preferred sites of PEGylation and then accurately predict conjugation rates and outcomes. Herein, we present a structure-dependent reactivity model that describes the rate of protein modification with amine-reactive PEG reagents to quantitatively predict lysine reactivity. In this model, we determine the sequence of reactivity by considering a distance-dependent metric for PEG shielding to explicitly reflect the effect of the site of modification on subsequent PEGylations. This model can aid with rapid optimization of both conjugate yield and specificity, enabling the development of PEGylated proteins in a data-driven, efficient, and streamlined manner (Figure 1).

## Results and Discussion

### A Distance-Dependent Metric for PEG Shielding

**Figure 2:**
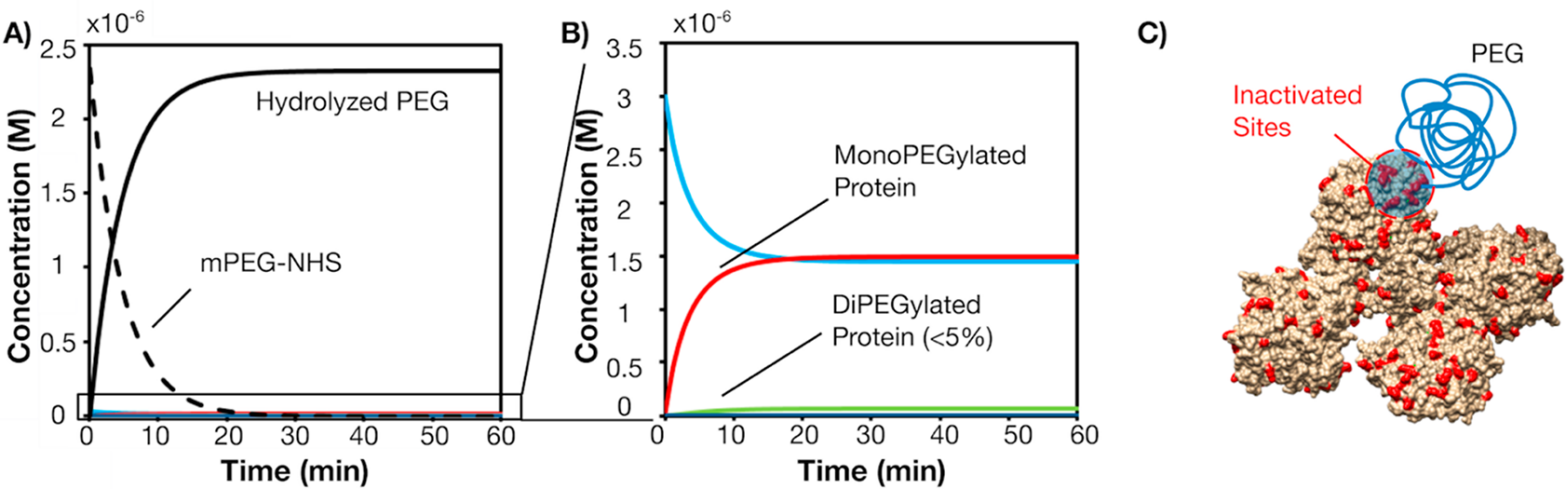
Time progression of BChE PEGylation with 5 kDa mPEG-NHS showing primarily mono-PEGylation. (A) Full PEGylation profile, black solid line: hydrolyzed PEG, black dashed line: mPEG-NHS and (B) enlarged profile of PEGylated BChE, blue line: native BChE, red line: mono-PEGylated BChE, green line: di-PEGylated BChE. (C) Depiction of local site hindrance from a PEGylated residue, lysine residues are colored in red. Model protein: BChE (PDB: 6I2T).

In a previous study, we successfully adopted the iso-reactivity model to determine amine PEGylation kinetics for butylcholinesterase.^16^ Our fitted parameters agreed with experimental findings, with monoPEGylated BChE being the dominant conjugate under the performed reaction conditions (Figure 2 A–B).

Upon closer analysis, we observed that the shielding parameter for butylcholinesterase (4.4 × 10^–4^ mol·g^–1^) was ∼10 times higher than the one determined by Pfister and co-workers (3.1 × 10^–5^ mol·g^–1^) for lysozyme (Table 1). PEG shielding, an effect that results from protein conjugation, can be viewed as a diminished exposed surface area for subsequent PEGylations. Once covalently attached, PEG (or any other polymer) can dynamically move around the protein surface, effectively masking available reactive sites and preventing them from conjugation. Hence, we hypothesized that the variation in shielding parameters were due to steric hindrance effects in proximal amine reactive sites. We considered shielding to be related to the radial distance from the amine reactive sites, where lysine residues near PEGylated sites are inhibited from subsequent PEG reactions (Figure 2C).

**Table 1:** Fitted Parameters for Lysozyme and BChE Using the Iso-reactivity Model^12^

	α (shielding)	κ (diffusion)	*k*_0_ (reactivity)
Lysozyme^12^	3.1 × 10^–5^	3.2 × 10^3^	10.7
BChE	4.4 × 10^–4^	7.2 × 10^3^	7.3

**Figure 3:**
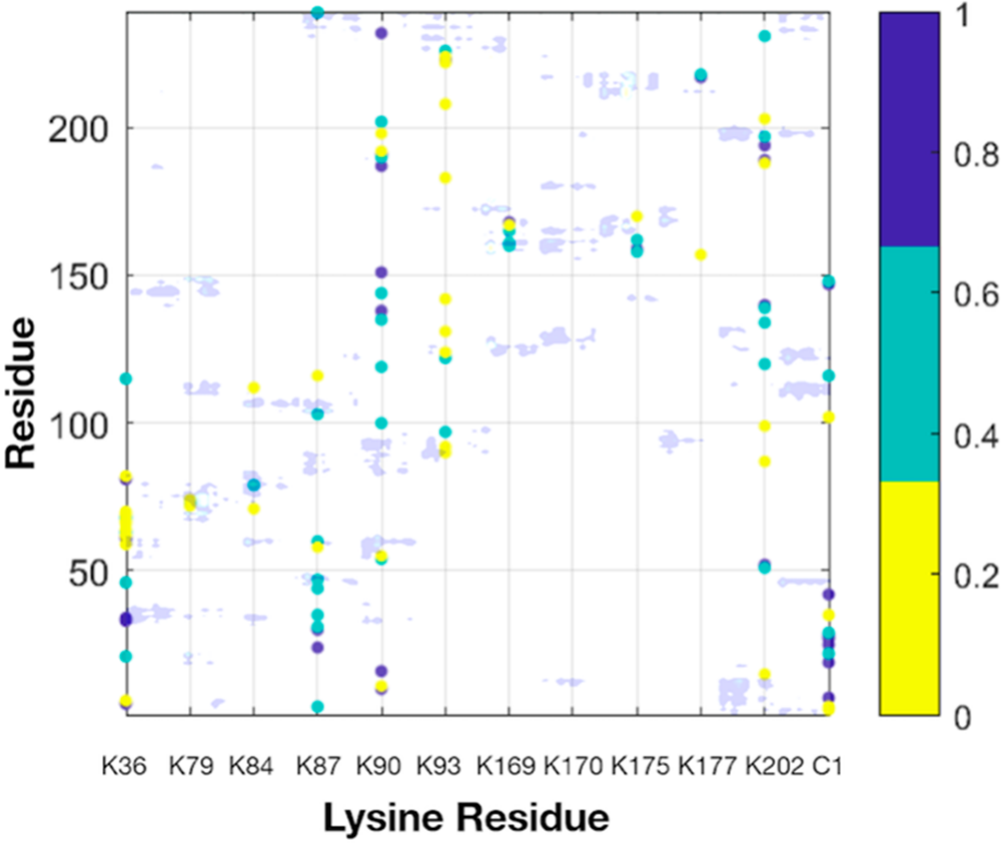
Contact map analysis for chymotrypsin plotted as a heat map of lysine residue versus all residues. The color intensity on the heat map corresponds to calculated Euclidean distances measured in Å. Inter-residue distances between 0–0.33, 0.33–0.66, and 0.66–1.0 Å are depicted in yellow, green, and blue, respectively. Light blue shade corresponds to contact residence time between pCBMA polymer and chymotrypsin reproduced from ref (17). Shorter inter-residue distances correlated with longer polymer-residue contact times, supporting the use of a distance-dependent metric for PEG shielding

To validate this assumption, we compared inter-residue distances for chymotrypsin with a contact map for chymotrypsin–poly (carboxybetaine) methacrylate conjugates (Figure 3).^17^ We noted that shorter inter-residue distances correlated with longer polymer-residue contact times, supporting our hypothesis of a distance-dependent metric for polymer shielding through steric hindrance. Although this analysis is based on zwitterionic poly (carboxybetaine) conjugates, amphiphilic PEGylated proteins also have strong interactions with protein surfaces through hydrophobic interactions.^18^ We therefore anticipate a similar correlation to be observed for PEGylated proteins.

### A Quantitative, Structure-Dependent Reactivity Model for Amine PEGylation

#### Estimation of Key Molecular Descriptors To Predict Amine Reactivity

Having established a distance-dependent metric for PEG shielding, we now sought to incorporate this parameter into a quantitative model to predict amine reactivity. Quantitative reactivity models are more beneficial for kinetic analysis and reaction optimization. However, determining the intrinsic reactivity of each residue is a time-consuming and laborious experiment and, as such, often restricted to smaller proteins.^4,12,19,20^ For larger multimeric proteins, such as butylcholinesterase with more than 100 amine reactive sites, obtaining experimental reactivity data is a challenging endeavor and can produce inaccurate results since not all modified sites can be validated experimentally.

To develop our quantitative model, we first wanted to identify which molecular descriptors were key to amine reactivity. Researchers, including ourselves, have noted that lysine residue reactivity can be profiled intuitively by assessing exposed surface areas (ESA) and amine p*K*_a_ values.^4,21−23^ This is not surprising since ESA reflects the accessibility of the amine group and p*K*_a_ the propensity of nucleophilic attack. However, our own structure–reactivity studies have also highlighted the contributions of local secondary structure and local surface charge. To gain further insight into the reactivity contributions of these molecular descriptors, two linear predictive models were developed.

Model 1 used ESA and p*K*_a_ as reactivity parameters, while Model 2 (like the PRELYM approach) incorporated an additional linear term for surface charge when lysine residues were in a β-sheet or coil fold. Both models were restricted to linear relationships between reactivity and molecular descriptors (ESA, p*K*_a_, surface charge). To account for potential nonlinearity, a machine-learned regression model (Model 3) was also developed. Machine-learning models are beneficial since they are not restricted to linear relationships and can model significantly more complex relationships between parameters. The drawback is the inability to draw physical information from the model, since the parameters within the model are black-boxed.

**Figure 4:**
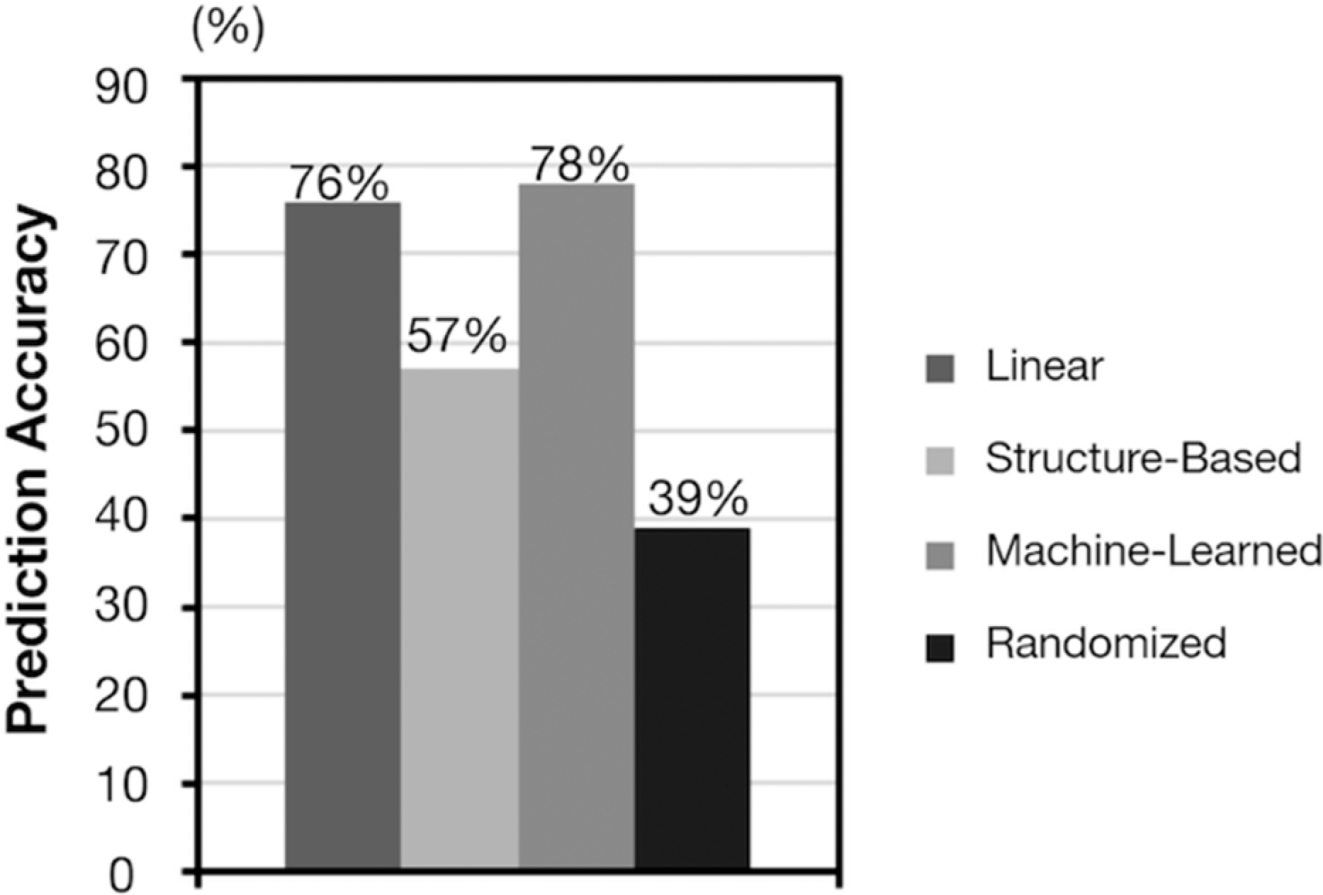
Linear, structure-based, and machine-learned models were assessed against the order of reactivity in BChE. A randomized model was used as a control. Prediction accuracy was defined as the number of correct predictions divided by the number of total trials.

Models 1, 2, and 3 were first standardized against experimental amine reactivity rates in lysozyme.^12^ Given the small number of amine sites in lysozyme, this fitting was performed with very few data points. To increase model reliability, we further trained the models using experimentally determined reaction orders in chymotrypsin.^4^ Model validation was achieved only when the predicted reactivity order correlated with experimental findings. The accuracy of the model’s performance was then assessed against the order of reactivity in BChE, as predicted using the tertiary-structure based decision tree (Figure 4). As a control experiment, we used a randomized model where the reactivities of BchE residues were randomly distributed.

Model comparison indicated that the linear model 1 achieved an accuracy of 76%, further highlighting the positive correlation between amine reactivity with amine ESA and p*K*_a_. The machine-learned model 3 showed a statistically insignificant higher accuracy, possibly as a result of Model 3′s ability to detect subtle nonlinearity. For example, lysine residues K79 and K170 in chymotrypsin are highly exposed (ESA > 200 Å^2^) with similar p*K*_a_ values. However, experimentally K79 had a low reaction rate and K170 did not react, suggesting deviation from a linear relationship.^4^ We believe that the reliability of the machine-learned Model 3 is being limited by the data extrapolation needed given available training data (lysozyme and chymotrypsin are both smaller than 25 kDa) being used to predict values for a large protein (BChE is approximately 270 kDa). Generating more training data is beyond the focus of this study, but the model would be improved by incorporation of experimental data with a more diverse range of features (e.g., molecular weight, quaternary structure) along with a more accurate depiction of protein structures in solution by solvation and relaxation using molecular dynamic studies. However, previous studies have shown that a significant improvement was not obtained when using descriptors calculated from annealed structures from MD,^21^ highlighting the challenge in obtaining complete accuracy in these predictive models.

The preferential interaction between PEG and lysine residues in hydrophobic surroundings, and the stabilization effect of PEG in α-helices, prompted us to consider hydrophobicity and helicity as additional features in our models.^24−26^ We observed that the association between inter-residue distance and contact time was slightly stronger when considering hydrophobicity and helicity with distance. However, an improvement in the prediction accuracy (∼70% accuracy after adding features) was not achieved and we could not establish a correlation between predicted reactivity with either hydrophobicity or helicity (Figure S1). This may be due to the close relationship between hydrophobicity and helicity with p*K*_a_, secondary structure, and local charge of lysine residues. Moreover, hydrophobicity and helicity are calculated by pooling empirically determined scores of surrounding residues but do not consider residues that are spatially close but separated in amino acid sequence.

#### Defining Parameters To Predict Subsequent PEGylation Sites

To incorporate our distance-dependent metric for PEG shielding, we selected the two best-performing models: linear model 1 and machine-learned model 3 using a positive linear activation function with two hidden layers and four nodes in each layer for amine reactivity prediction. It should be noted that our model can only be employed for biologically relevant conditions (pH 7–8, for example) as we do not consider protein structural changes such as unfolding, which can occur at extreme pH conditions. For predictions outside of this pH range, we suggest the use of molecular dynamic simulations to account for protein unfolding along with model reparametrization since features such as Coulombic charge used in our model are pH specific.^4,12^

Having the tools to predict the lysine reactivity, we now sought to define parameters to predict the sequence in which an individual lysine would be modified by a molecule that could then shield the surface from further modification. Once a PEG chain is attached to a protein, it adopts either a “dumbbell” shape extending from the site of conjugation to the solvent or a “shroud” conformation that wraps around the protein, making shielding sites less predictable.^27^ Since PEGs below 10 kDa will predominantly adopt a dumbbell shape, our model incorporated this assumption although the exact conformation will depend on the specific protein and site of conjugation.^18,25,27,28^ Our model further makes the assumptions that the protein structure remains unchanged upon PEGylation, and the reaction mixture is sufficiently dilute to uphold the underlying diffusion regime.^29−31^

**Scheme 1:**
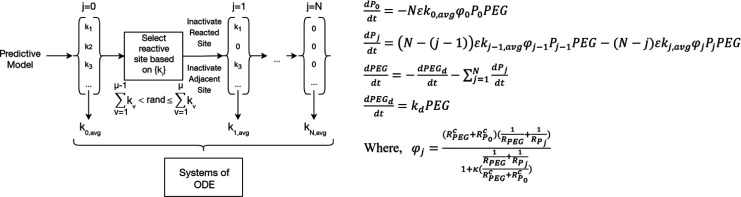
Kinetic Constant Estimation for Subsequent PEGylation (Left); Key Ordinary Differential Equations (ODEs) of the Kinetic Model (Right)^12^

We defined a radial distance cutoff where lysine residues within this radius will be nonreactive due to steric hindrance (Scheme 1, Figure 2C). Physically, this parameter is comparable to the radius of PEG coverage but differs from the radius of gyration, since PEG coverage can be affected by many factors including the shape of the PEG chain and interactions with the protein surface. From here on, only results from a single simulation are shown, while the stochastic nature of multiple simulations can be seen in Figure S2.

#### Model Validation with PEGylated Proteins

To confirm that our structure-dependent reactivity model would generate acceptable PEGylation reactivities, we tested models 1 and 3 using three proteins: human growth hormone receptor antagonist (Somavert, pegvisomant), interferon α-2a (PEGASYS), and recombinant phenylalanine ammonia lyase. These three proteins have been PEGylated and are well characterized in terms of the number and site of modifications.

### Human Growth Hormone Receptor Antagonist

Pegvisomant (trade name Somavert) is a Food and Drug Administration (FDA) approved human growth hormone receptor antagonist for the treatment of acromegaly.^32^ Pegvisomant is covalently modified with a 5 kDa mPEG-succinimidyl propionate at pH 7.65 and therefore follows the underlying assumptions in our model.^57^

Reactivity predictions using linear and machine learning models both matched experimental findings, with five amino sites being modified (N-terminal, K38, K120, K140, and K158) (Table 2). Noticeably, linear model 1 predicts K115 as a reactive site, albeit with low reactivity while machine learned model 3 does not, further emphasizing its improved accuracy and possible detection of nonlinearity in reactivity. Neither model predicted K120 as a reactive site; however, this residue has been mutated in pegvisomant and is not present in the protein crystal structure used for this analysis.^33^ Noteworthy, upon K120 modification, the close spatial proximity to K115 suggests further PEGylation reactions would be blocked by steric hindrance, which aligns with our current predicted results.

**Table 2:** Comparison of Experimental Findings for PEG Modification of Somavert^34^ with Reactivity Predictions Using Linear and Machine-Learned Models

		Linear Model 1		Machine Learned Model	
Residue	Experimental	Modification	Reactivity Rate (M^–1^·min^–1^)	Modification	Reactivity Rate (M^–1^·min^–1^)
N-terminal	×	×	13.06	×	19.76
K38	×	×	10.97	×	16.36
K41					
K70					
K115		×	6.04		
K120	×	N/A	N/A	N/A	N/A
K140	×	×	12.38	×	20.55
K145					
K158	×	×	4.77	×	6.03

### Interferon α-2a and Recombinant Phenylalanine Ammonia Lyase

We further expanded our model validation to interferon α-2a and recombinant phenylalanine ammonia lyase (rAV-PAL). PEGylated interferon α-2a, commercially known as PEGASYS, is formulated with a 40 kDa branched PEG chain^3^ while rAV-PAL is modified with PEG 20 kDa.^35^ Due to probe size constraints, reactivity predictions for both proteins were kept at a probe size equivalent to 20 kDa PEG. Nevertheless, our structure-dependent reactivity predictions were in good agreement with the experimental findings (Tables S1 and S2).

Interestingly, for rAV-PAL, the linear model performed better, with a Pearson correlation coefficient of 0.64 between the experimentally observed degree of PEGylation and predicted reactivity. Amine sites K109 and K384 were inaccurately predicted as modified, but with low reactivity rates. K384 is in close proximity to K335 (Euclidian distance = 8.85 Å) which is an experimentally determined PEGylation site and can hinder K384 subsequent modification.

Similarly, for interferon α-2a, the N-terminus and K23 were predicted incorrectly as reactive sites but with low reactivity rates. This can be also attributed to the use of a smaller probe size, due to software constraints, which may have increased the apparent ESA, thus increasing the predicted reactivity. We should also highlight that the available PDB file for interferon α-2a consists of 24 NMR-resolved structural conformers, and only a single conformer was used for the prediction. Protein solvation quality may result in dynamic changes in conformation, releasing constrained sites and increasing ESA. Improved quantitative predictions can therefore be obtained by using more accurate solvated structures, obtained for example, through molecular dynamics simulations to achieve the lowest energy conformation.

### Predicting Site-Specific “PEGmer” Distribution and PEGylation Reaction Progress

One of the major challenges in PEGylation is the ability to produce the desired PEG-protein conjugate at high yields. In recent years, extensive research efforts have been made on developing novel PEGylation reagents and conjugation chemistries to improve the efficacy of these chemical reactions.^36,37^ However, for amine-targeted PEGylation, the multiplicity of conjugation sites can result in complex and heterogeneous mixtures of distributed populations of PEG-protein conjugates, with various grafting densities, and PEG positional isomers. Isolation of the desired bioconjugate will then require supplementary purification steps which can reduce conjugate yield and impact the cost of the final product. Moreover, each unique PEG-protein conjugate, also known as a “PEGmer”, can display variable bioactivity, and pharmacokinetic properties.^3,38^ Characterization of the various PEGmers is therefore necessary to understand therapeutic efficacy and often a requirement for regulatory approval.^39^ Kinetic reactivity models can help resolve experimental observations and facilitate bioconjugation optimization, minimizing developmental costs. To demonstrate these advantages, we used our validated structure-dependent reactivity model to gain further insight into the PEGylation reaction.

#### PEGmer Distribution

**Figure 5:**
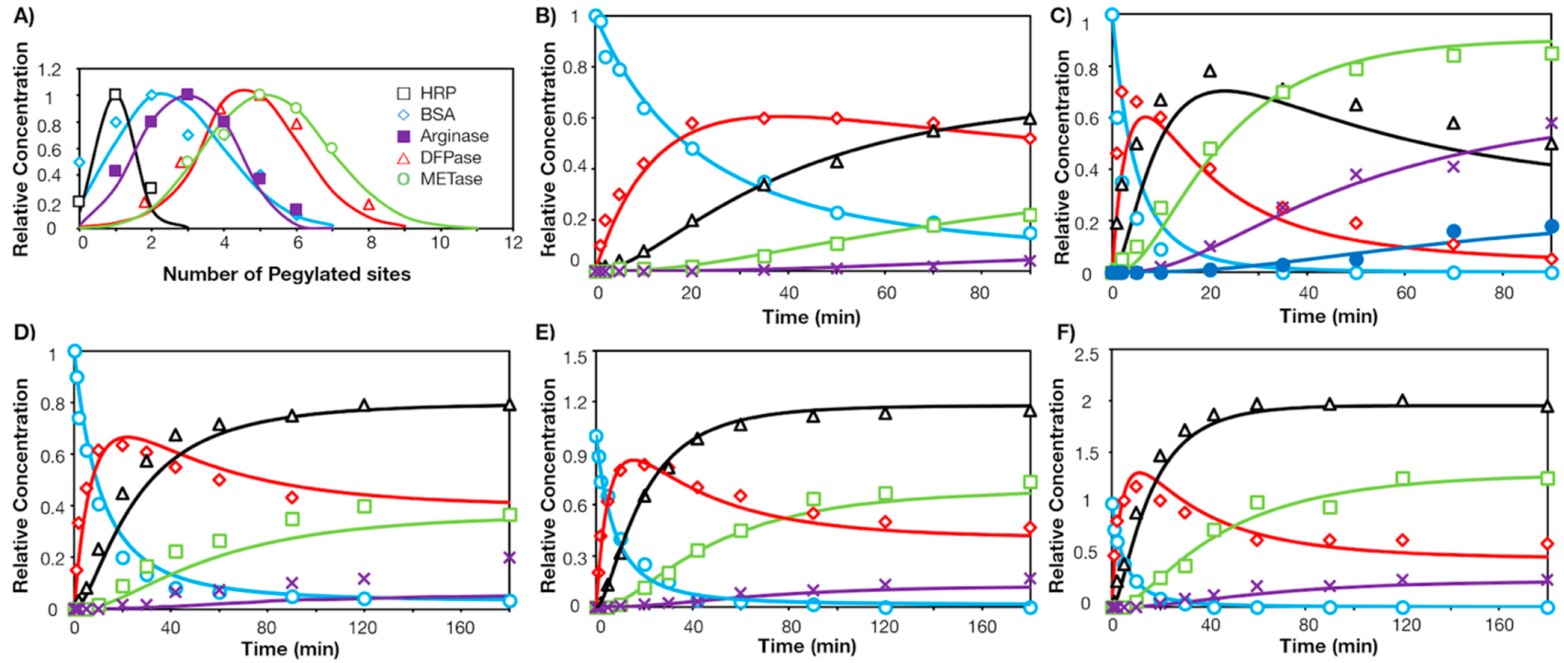
Comparison between model simulation and literature data. (A) Distribution of PEGmers for horseradish peroxidase (HRP), bovine serum albumin (BSA), human arginase, diisopropyl fluorophosphatase (DFPase), and methioninase (METase). Reaction progress curves of α-lactalbumin with (B) mPEG-SMB and (C) mPEG-SPA. Reaction progress curves of lysozyme with mPEG-SPA and constant ESA calculated with a 10 kDa PEG probe (17 Å) for (D) 5 kDa, (E) 10 kDa, (F) 20 kDa PEGylation. Reactions are simulated with respective experimental conditions.^12,40−45^ Symbols represent literature values, and solid lines represent simulated values. In B–F, (○) Native Protein, (◇) PEG-1, (△) PEG-2, (□) PEG-3, (×) PEG-4, (●) PEG-5. Relative concentration was calculated by normalizing against the initial mass concentration of the native protein.

To simulate the PEGylation reaction, we selected linear model 1 due to its simplicity and high prediction accuracy. After fitting to optimal parameters, we now wanted to obtain detailed information on the extent of PEGylation, the distribution of the various PEGmer populations, and respective amine modifications. Figure 5A compares experimental findings with our simulated PEGylation reactions using horseradish peroxidase (HRP), bovine serum albumin (BSA), human arginase, diisopropyl fluorophosphatase (DFPase), and methioninase (METase). Overall, our model was able to accurately predict PEGmer distribution for all examined proteins.

These results indicate that our model can identify ideal reaction conditions for optimal PEGylation, to ensure high conversion and purification yields of the desired conjugate. Since the reaction site with the highest predicted reactivity is not always the first to react, and rather reaction order follows a probability distribution, the stochastic nature of reaction can be included (Figure S2B) and fine-tuned to select for reaction conditions producing highly uniform PEGylated proteins. By merging our structure–reactivity predictions with kinetic modeling, we were also able to monitor the time progression of site-specific PEGmers (Figure S3A). Combined with our previously devised amine-reactive inhibitor, which can be used to selectively quench *fast-reacting* sites, we can further exploit reaction conditions to achieve highly flexible conjugation.^46^ Moreover, our structure-dependent reactivity model can be used alongside other predictive algorithms to synthesize protein conjugates with a tailored activity and stability profile in a structured manner without the need for extensive trial-and-error experiments.^12,40^

#### Simulated PEGylation Reaction Curves

PEGylation reaction curves were simulated for the lysozyme, α-lactalbumin, and antibody single-chain variable fragment (Figure 5B–F, Figure S5), with low root-mean-square error (RMSE). To account for the influence of different reactivity due to PEG chemical reactivity, molecular weight, and reaction conditions (e.g., buffer, pH, temperature, etc.), we included a prefactor to the predicted reactivity (Table 3).

**Table 3:** Parameters Corresponding to Figure 5A^a^^,^^b^

Protein	PEG Mw (kDa)	PEG linker	κ (×10^–3^, Å^2^)	Reaction Cut-off (Å^2^)	Reaction Prefactor	Experimental RMSE	RMSE^12^
**LALBA**	5	SMB	6.3	16	29	0.02	0.1
	5	SPA	6.5	17	143	0.06	0.06
**LYZ**	5	SPA	2.8	<11 (<11)	100 (150)	0.06 (0.04)	0.08
	10	SPA	2.8	<11 (<11)	100 (100)	0.04 (0.04)	0.1
	20	SPA	2.8	<11 (15)	100 (79)	0.1 (0.1)	0.1

a Values in brackets reflect changes in ESA from different probe sizes corresponding to different PEG molecular weights.

b Parameters were fitted for each protein according to respective reaction conditions. The threshold for reaction cut-off was optimized to 11 Å, since it is approximately the smallest Euclidian distance between lysine residues in lysozyme. PEG coverage below 11 Å is considered insignificant.

Figure 5 B and C depict the PEGylation reaction curves for α-lactalbumin with succinimidyl esters of methoxy poly(ethylene glycol) α-methyl butanoic acid (mPEG-SMB) and propanoic acid (mPEG-SPA). PEG-shielding in α-lactalbumin using both SPA and SMB linkers with 5 kDa PEG chains showed consistent results (16 and 17 Å PEG coverage), confirming PEG shielding was independent of the reactive group chemistry. The lower reactivity of mPEG-SMB is accurately predicted, as can be seen by the incomplete conversion of native α-lactalbumin over time. This highlights our model’s applicability to different PEG chemistries, where the reaction prefactor can sufficiently address the difference in linker reactivity, dissimilar to earlier PEGylation kinetic models (Figure S6).^40^ We also observe the reactivity of mPEG-SPA is similar in lysozyme and α-lactalbumin through similar reaction prefactors in 5 kDa PEGylation reactions (Table 3, Figures S3–S4). This suggests lysine-NHS ester reactivity is retained between different protein structures.

Moreover, we note that our model can be successfully implemented even with the same set of parameters for different PEG chain lengths (5, 10, 20 kDa, Figure 5 D–F, Table 3, Figures S3–S4). This demonstrates an added benefit that only three parameters (diffusion, shielding, and reaction prefactor) need to be optimized, expanding the model generalizability, and reducing computational cost. However, to adequately incorporate in the model the changes in ESA due to different probe sizes (and consequently PEG molecular weights), the reaction prefactor needs to be adjusted for the predicted reactivities. Although this slightly reduces model flexibility, since unique parameters are now required to be fitted for each probe size, this allows us to unravel when the PEG shielding effect becomes important (Table 3, Figures S3–S4). Indeed, PEGylation with a 20 kDa polymer leads to an emphasized shielding effect, determined by the unreactive sites 15 Å from site of PEG modification. In contrast, for 5 and 10 kDa PEG chains, the reaction coverage is <11 Å, and thus, no significant shielding constraints were imposed. Here, the value of 11 Å was considered the threshold for reaction cutoff, as this was the smallest Euclidean distance calculated between two adjacent lysine residues in lysozyme. Thus, any shielding effect smaller than 11 Å was not thought to impact reactivity. Lastly, the increasing degree of shielding from lysozyme, α-lactalbumin, to single-chain variable fragment (scFv) can be explained by the increasing hydrophobicity of the protein surface (Figure S7).

It is interesting to note that our model underestimated the formation of a tetra-PEGylated lysozyme conjugate using PEG 5 kDa (Figure 5 D, Figure S3). This observation suggested that there may be a change in polymer conformation on the protein surface at high grafting densities that was dependent on PEG size. We hypothesized that when the PEG grafting density was high, longer PEG chains may have experienced more repulsion and were more extended toward the surrounding solvent, consequently reducing PEG coverage on the protein surface, allowing the exposure of more reaction sites. The conformational transition from a *dumbbell* to *brush* conformation at high grafting density has been thoroughly studied on surfaces, where the change in conformation is remarked by the relation between the distance between two grafts (*D*) and a polymer size-dependent Flory radius (*R*_f_).^47^ However, experimental evidence of conformational changes at high degrees of PEGylation on protein surfaces has been scarcely observed, with reports limited to diPEGylation,^48−50^ or simple globular structures such as nanoparticles,^51,52^ although high density PEGylation has been achieved in lysozyme.^53^ Recently, high-density PEGylation of ovalbumin has been reported,^54^ with specific references toward a brush-like conformation at *R*_f_/*D* > 2. In a similar manner, we estimated an *R*_f_/*D* ratio of 1.47 and 2.22 for tetra-PEGylated lysozyme for a PEG length of 5 and 10 kDa, respectively (see Supporting Information for calculation details). This corresponded to a dumbbell-to-brush transition, as observed by the low degree of tetra-PEGylated lysozyme with the 5 kDa PEG chain (Figure 5D–F). Although lysozyme is a simple model protein, our reaction model provides an innovative way of examining polymeric conformational changes on the surface of PEGylated proteins.

## Conclusions

In this study, a structure-dependent reactivity model that introduced the radius of PEG chain coverage as a tangible, structure-specific shielding parameter was developed. This structure-dependent reactivity was implemented in three models to unravel how protein-specific molecular descriptors can shed light on the relative reactivity of lysine residues. Model accuracy was found to be over 75% in the prediction of BChE reactivity order using linear and machine-learned models. Further validation with PEGylated proteins showed a good correlation between predicted and experimentally determined degree of modification. Application of our model to simulate PEGylation progress curves and estimate site-specific PEGmer distribution led to accurate predictions with an on average 35% reduction in RMSE from the original model across different proteins, PEG linkers, and molecular weights. Moreover, this analysis provided us with unique insights into PEG conformational changes on highly grafted protein surfaces as a function of PEG molecular weight.

While our model has been trained for protein PEGylation, application can be extended to different polymers or small-molecule initiators by reparametrization, emphasizing its wider benefit to the bioconjugation field. Moreover, future work on temperature and pH dependency of the model particularly can further enhance the model’s generalizability by fitting the intrinsic rate constant through an Arrhenius-like relationship. Overall, implementation of this reactivity model has the potential to help develop and design experimental work in a more efficient and cost-effective manner.

## Experimental Procedures

### Inter-residue Distance Calculations

Protein crystal structures were retrieved from the Protein Data Bank (PDB) (Table 4). PDB coordinate files were loaded in Matlab 2019, and inter-residue distances, calculated as Euclidean distances. Lysine-lysine residue distances were calculated as the distance between ε-amines (denoted as NZ in PDB coordinate files); distances between a given lysine and other residues were calculated as the distance between the ε-amine group and the α-carbon (denoted as CA in PDB coordinate files) of each residue.

**Table 4:** Proteins Used in This Study with Respective PDB ID and Number of Available Amine Reactive Sites

Protein	PDB ID	Number of amine reactive sites
Lysozyme	1LYZ	6–7
Chymotrypsin	4CHA	15
Butylcholinesterase (BChE)	6I2T	136
Bovine Serum Albumin (BSA)	4F5S	60
Diisopropyl Fluorophosphatase (DFPase)	1PJX	25
Horseradish Peroxidase (HRP)	1H57	7
Methioninase (METase)	1UKJ	33
Human Arginase	1WVA	23
Human Growth Hormone	3HHR	10
Α-lactalbumin	1A4 V	12
Single Chain Variable Fragment (scFv)	6PIL	10
Tetrameric phenylalanine ammonia lyase (rAV-PAL).	5LTM	16^a^
Interferon-a 2a	1ITF	8^a^

a Original proteins possess 19 reactive sites (rAV-PAL) and 10 reactive sites (interferon-a 2a), respectively. Calculated reactive sites were reduced to 16 and 8, respectively, due to missing residues in the PDB structure.

### Estimation of Individual Lysine Exposed Surface Area, p*K*_a_, Surface Charge, Hydrophilicity, and Helicity

#### Exposed Surface Area (ESA)

The exposed surface area for each residue was calculated with UCSF Chimera^55^ using a Shrake–Rupley (“rolling ball”) algorithm. Probe sizes were selected to resemble the approximate size of PEG 5, 10, or 20 kDa, or an atom-transfer radical polymerization (ATRP) initiator molecule (4.2 Å).^4^ Probe sizes for PEG were estimated by respective radii of gyration (*R*_g_) assuming freely jointed chains (12–24 Å).^4^ For large proteins (≥60 kDa), a maximum probe size of 8.8 Å was used. Calculated ESA followed an approximately linear relationship with the various probe sizes (Figure S8).

***pK*_*a*_*.*** The p*K*_a_ of protein residues was calculated using H++ at experimental pH 8 with continuum electrostatics.^56,57^ A protein dielectric constant of 10, water dielectric constant of 80, and salt concentration of 0.15 M were used for the calculation. For proteins with missing residues, H++ could not be used and thus PROPKA was used alternatively for empirical p*K*_a_ calculation.^58,59^

#### Residue Surface Charge, Hydrophobicity, and Helicity

The Coulombic charge (kcal·e^–1^·mol^–1^) of protein residues was calculated with UCSF Chimera, and the charge for lysine ε-amino groups was recorded. Residue hydrophobicity and helicity were calculated using ExPASy ProtScale with Kyte & Doolittle and Levitt scales, respectively. The Kyle and Doolittle scale considers both structural contributions from the lysine side chain group and its interaction with water to determine the hydropathy index. The Levitt scale calculates helicity by measuring the frequency of amino acid occurrence in an α helix. ProtScale calculations proceed through a sliding window technique which weighs the scores of seven residues with the residue in question assigned to the center with a 100% weight, which linearly decreases to 10% for the outermost residue.^60−62^

### Linear, Tertiary Structure-Based, and Machine-Learned Model to Predict Individual Amine Reactivity

Three models (linear, tertiary structure-based, and machine-learned) were developed to predict the rate of PEGylation at each lysine residue. Linear and structure-based models are detailed as below:

**Linear model:**
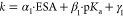
1

**Tertiary structure-based model:**
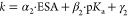
2

when β-sheet and coil,
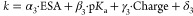
3where α, β, γ, and δ are regression parameters and the subscripts denote different coefficients for each model. When residues are in a β-sheet or coil fold, the tertiary structure-based model incorporates an additional linear term for surface charge due to their closer proximity to ionizable groups in the protein, as previously determined.^4^ The coefficients of each model were fitted with intrinsic reaction rates for lysozyme and molecular descriptors as detailed above. The model fitting optimization was run 1,000 times, and the parameters corresponding to the minimum least-squares difference between literature intrinsic rates and regressed reaction rates were recorded. The model fits obtained were α_1_ = 0.06, β_1_ = −1.32, γ_1_ = 15.02, α_2_ = 0.087, β_2_ = −1.61, γ_2_ = 17.16, α_3_ = 11.97, β_3_ = −6.89, γ_3_ = −14.25, and δ_3_ = 17.16, with *R*^2^ for the linear, structure-based, and machine-learned models as 0.85, 0.94, and 0.93, respectively. The models are denoted as follows: linear model (Model 1), tertiary structure-based model (Model 2), and machine-learned model (Model 3).

A feed-forward model was built using the Statistics and Machine Learning Toolbox in Matlab 2019. Experimental kinetic rate constants for lysozyme reacting with 5 kDa PEG and respective calculated descriptors were used to train the model.^12^ Since lysine residues in lysozyme are primarily found in the α-helix fold of the protein, a hold-out method was first used to validate the prediction for residues with α-helix secondary structures to prevent prediction against unseen data. The trained model was then used to predict reaction rates of chymotrypsin with an ATRP initiator molecule (4.2 Å). Predicted reaction rates were sorted in descending order into *fast-reacting* (3 out of 10 sites), *slow-reacting* (4 out of 10 sites), and *nonreacting* (3 out of 10 sites), as determined experimentally.^4^ The trained model was validated only if the predicted reaction order matched experimental data.

To assess how the three models in this study compared to our PRELYM decision tree^4^ model, a test set composed of 136 lysine residues and N-termini present in butyrylcholinesterase (BChE) with 5 kDa PEGylation was built. We stress that PRELYM was built for small molecule modification of lysine residues. This test set was first predicted qualitatively using the tertiary structure-based decision tree model.^4^ Molecular descriptors (ESA, p*K*_a_, residue surface charge, hydrophobicity, and helicity) of BChE were then used to quantitatively predict the reactivity of each amino site, and reactivities were sorted from fast to slow and categorized into *fast-* (16 out of 136 residues), *slow-* (43 out of 136 residues), and *nonreacting* (77 out of 136 residues) to match that of the tertiary-structure based decision tree. The accuracy of the quantitative model was assessed by the number of correct predictions divided by the total number of reactive sites. Further, a “reactivity cutoff” parameter was used to define the highest predicted reactivity that is categorized as “nonreactive” in the decision tree. This cutoff is used subsequently to render sites with a predicted reactivity below this value to be nonreactive.

### Structure-Dependent Reactivity Model and Parameter Fitting

Scheme 1 depicts the characteristics of the ‘structure-dependent reactivity model’ developed in this study. This model was first used to identify the relative intrinsic reactivities of lysine residues and the *N*-terminus with a reactivity cutoff of 3.9 M^–1^·min^–1^ using predictive models 1–3. The average of the predicted reactivities was used to represent the reactivity for the current PEGylation reaction. It should be noted that although raw, unaveraged kinetic constants can be used to monitor reaction progression,^12^ the average value was used to reduce computational time while still producing physically sound kinetic constants.

For subsequent PEGylations, since the highest predicted reactivity was not guaranteed to react, we introduced stochasticity by the Gillespie-like algorithm, which was used to select the reaction site through eqs 4–5.^63^ Here, *k*_v_ is the cumulative probability of reaction of a reactive site v, defined as the cumulative predicted reactivity of each site divided by the sum of reactivities from all sites. Thus, *k*_v_ is a numerical value between 0 and 1. By generating a random number “rand”, the algorithm can then select a reactive site μ with a probability proportional to its reactivity. Since the ratios between intrinsic kinetic constants match well with the concentration of PEGmers (Figure S9), we only considered intrinsic reactivity as a defining factor for site selectivity.
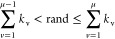
4
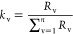
5

In the kinetic part of the model (Scheme 1, right), *N* is the total number of reactive sites, *P*_*j*_ is the molar concentration of *j*-pegylated protein (*j*∈[0,*N*]), *k*_*j*,avg_ is the intrinsic reactivity for the *j*-th PEGylation reaction, and ε is the reaction prefactor that modifies for PEG linker chemistries and reaction conditions. φ_j_ is a modifier for diffusional (κ) effects. ε*k*_*j*,avg_φ_j_ is then representative of the observed reactivity of the *j*-th PEGylation. PEG, PEG_d_, and *k*_d_ are the concentrations of methoxy poly(ethylene glycol) *N*-hydroxysuccinimide (mPEG-NHS), hydrolyzed mPEG-NHS, and the hydrolysis rate constant, respectively. The hydrolysis rate constant is 0.21 min^–1^ for mPEG-NHS and ∼0.01 min^–1^ for succinimidyl esters of methoxy poly(ethylene glycol) propanoic acid (mPEG-SPA) and α-methyl butanoic acid (mPEG-SMB).^12,40,64^ The hydrolysis rate constants for other PEG linkers were approximated from literature.^65,66^ The set of *N* + 3 differential equations were solved with ode15s on Matlab 2019. The model fitting optimization was run 50 times, and the parameters (κ, reaction prefactor, distance cutoff) corresponding to the minimum root-mean-square error (RMSE, eq 6) between literature values and simulated values were recorded. *C*_ex_ and *C*_calc_ denote the experimental and simulated concentrations, and *n* denotes the number of experiments.
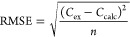
6

The 95% confidence interval for fitted parameters is constructed based on *F*-test statistics.^67^ Briefly, the 95% F-test score was calculated with degrees of freedom *n*_p_ and *n*, where *n*_p_ is the number of parameters to be fitted and *n* is the number of experiments. The confidence interval was calculated with eq 7, where *q* corresponds to the (1 – *q*) quantile of the F-distribution, **p** is the set of optimizable parameters, and p* is the best fit set of parameters. *D*(**p**) and *D*(p*) are RMSE values calculated from sets **p** and p*, respectively. Random noise was then induced to the optimized parameters p* to generate **p**, which are used to calculate the updated RMSE value *D*(**p**) between simulated and experimental data. The range of parameters that give RMSE values within the 95% confidence interval is then recorded.
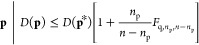
7

## Supporting Information Available

Effect of hydrophobicity and helicity on contact time; Analysis of lysozyme PEGylation, progress curves and respective fitted parameters; scFv PEGylation and respective fitted parameters; Simulation of α-lactalbumin with mPEG-SMB using Pfister model; Hydrophobicity scores of model proteins; Effect of different probe sizes on lysozyme ESA; PEG positional isomer concentrations and intrinsic rate concentrations; Reactive sites comparison for rAV-PAL and interferon-α 2a; Normalization of protein concentration for PEGylation simulations and calculation of PEG conformation on lysozyme. (PDF)

## Notes

The work described herein was performed at Carnegie Mellon University.

## Notes

Data and Software Availability: The data underlying this study are available in the published article and its online Supporting Information.
