# Supplementary material for: Moving Protein PEGylation from an Art to a Data Science
Source: Bioconjug Chem. 2022 Aug 22;33(9):1643–53. doi: 10.1021/acs.bioconjchem.2c00262 (PMC9501918; doi:10.1021/acs.bioconjchem.2c00262)
Supplement: Supplementary file 1 — bc2c00262_si_001.pdf [file bc2c00262_si_001.pdf]

Supporting Information

Moving Protein PEGylation from an Art to a Data Science

Leran Mao[1], Alan J. Russell[2], Sheiliza Carmali[3*]

[1] Department of Chemical Engineering, Carnegie Mellon University, Pittsburgh, Pennsylvania 15213, United States

[2] Amgen Inc., Thousand Oaks, California 91320, United States

[3] School of Pharmacy, Queen's University Belfast, Belfast BT9 7BL, United Kingdom

*Corresponding author:

E-mail: s.carmali@qub.ac.uk (SC)

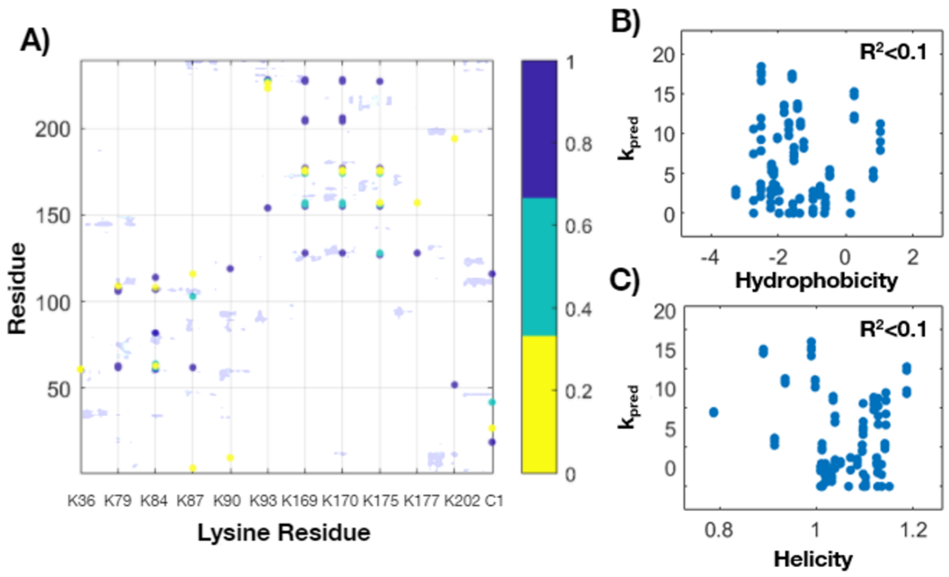

Figure S1. The effect of hydrophobicity and helicity on contact time. A) Contact map between conjugated lysine residues and all residues in chymotrypsin. The quantitative scoring is based on normalized $\frac{\text{Hydrophobicity} \cdot \text{Helicity}}{\text{Inter−residue Distance}}$. Scatter plot between predicted reactivity and B) hydrophobicity and C) helicity. Light blue shade corresponds to contact residence time between pCBMA polymer and chymotrypsin.[2]

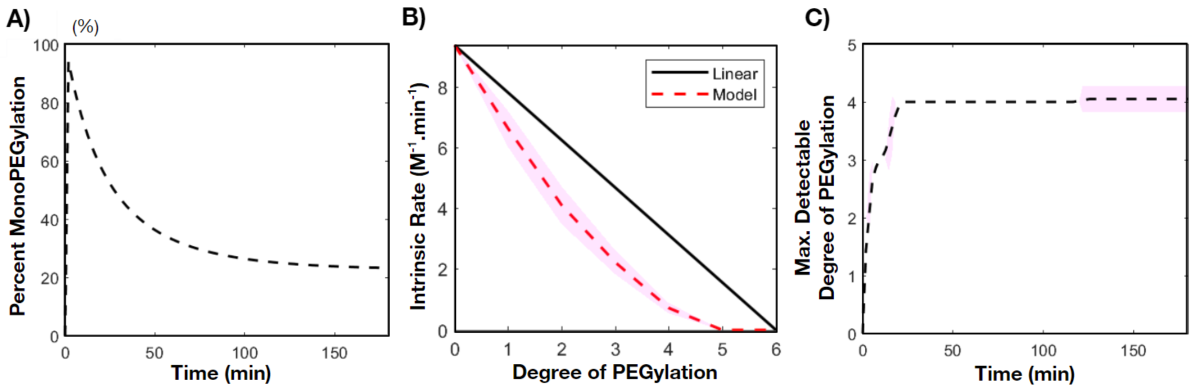

Figure S2. Analysis of PEGylation using 5kDa mPEG-NHS conjugation with lysozyme as an example. A) Percent of monoPEGylated conjugates at different stages of the reaction, B) Net effect of shielding on the rate of subsequent PEGylations, with stochasticity added through a Gillespie-like algorithm (Scheme 1). The model curve is shown as an average from 20 simulations. The linear reference represents model outcome without considering the reduction of reactivity through shielding, C) Max observable degrees of PEGylation of conjugates in the reaction mixture. The black dashed line is an average of 20 simulations. The magenta shade represents a standard deviation from 20 simulations. Conjugate is defined as observable if its concentration is higher than 0.1 % of the initial protein concentration.

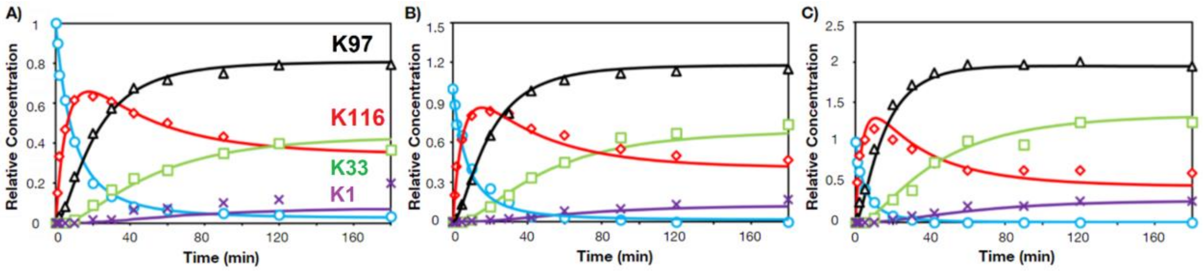

Figure S3. PEGylation progression of lysozyme with A) 5 kDa, B) 10 kDa, and C) 20 kDa mPEG-SPA. ESA is calculated with respective PEG sizes. (○) Native Protein, (◇) PEG-1, (△) PEG-2, (□) PEG-3, (X) PEG-4. Relative concentration was calculated by normalizing against the initial mass concentration of the native protein.

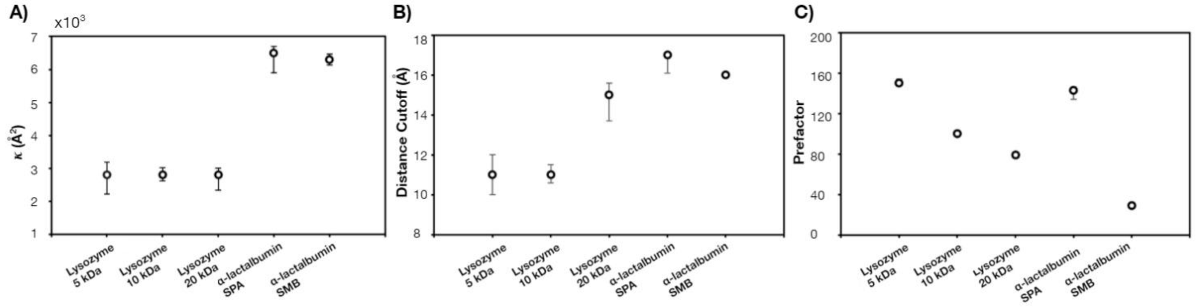

Figure S4. 95 % Confidence interval of fitted parameters. A) κ, B) distance cut-off, C) reaction prefactor. Lysozyme is simulated with respective probe sizes.

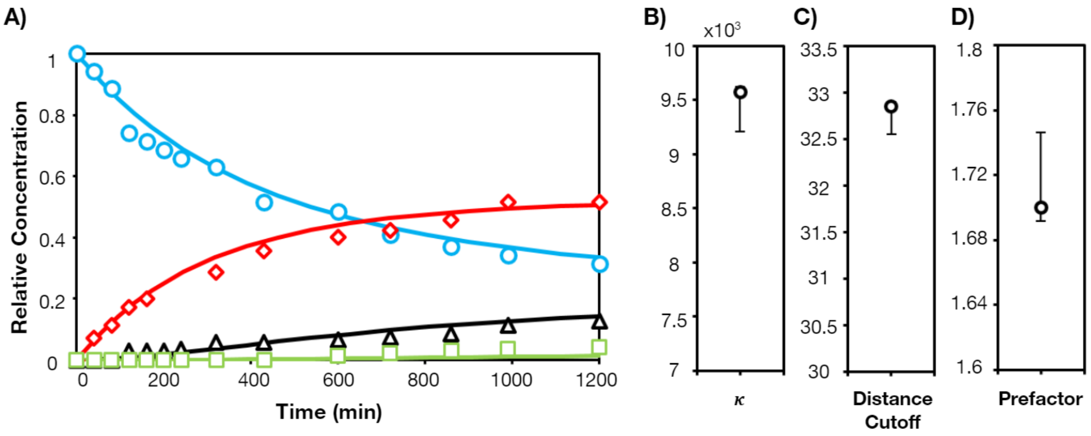

Figure S5. scFv PEGylation and respective fitted parameters. A) Reaction progression of scFv with 5kDa mPEG-NHS. Fitted parameters and respective 95 % confidence interval of B) κ, C) distance cutoff, and C) reaction prefactor. (○) Native Protein, (◇) PEG-1, (△) PEG-2, (□) PEG-3. Relative concentration was calculated by normalizing against the initial mass concentration of the native protein.

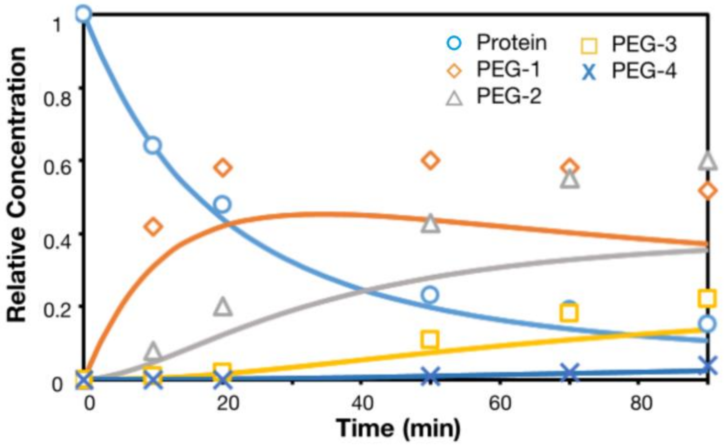

Figure S6. Simulation results with mPEG-SMB modification of α-lactalbumin using the Pfister model[1], showing slight discrepancy of model outcomes with a different PEG linker chemistry. The respective model parameters are: κ =0.0013 A^2, α=4.3x10^{-5} mol/g, k_0=41.34.

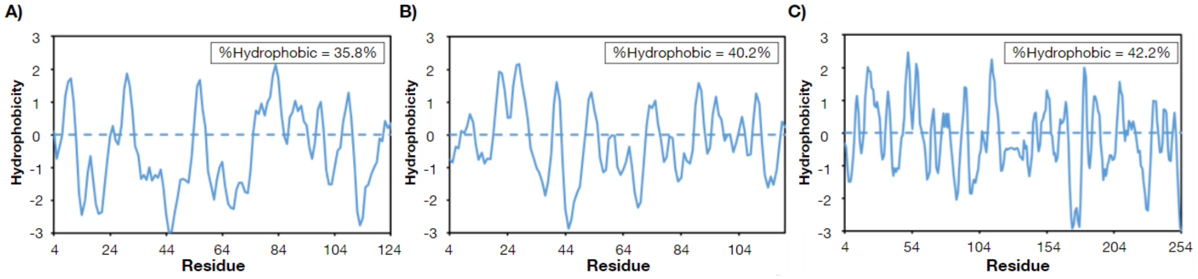

Figure S7. Hydrophobicity scores of model proteins A) Lysozyme, B) α-lactalbumin, C) scFv. Showing the trend lysozyme < α-lactalbumin < scFv with increasing hydrophobicity. The score of 0 is defined as the threshold between hydrophilicity and hydrophobicity, with hydrophobic residues having a score >0.

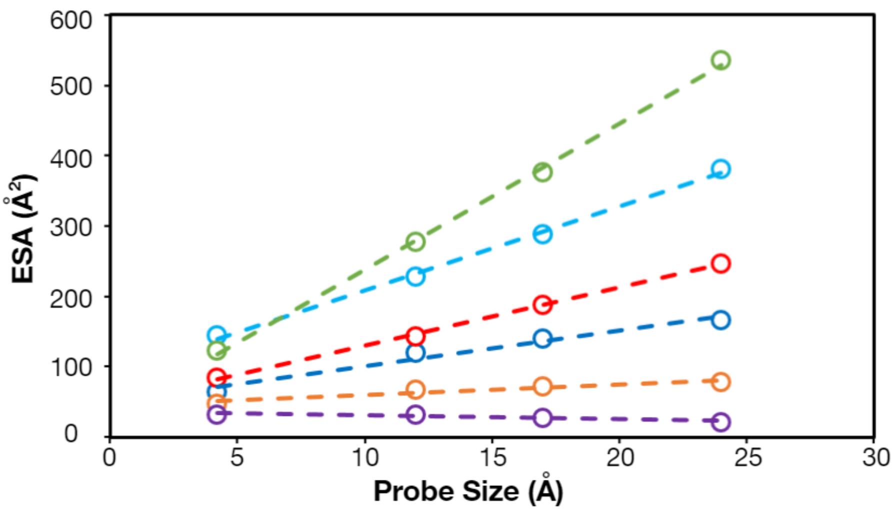

Figure S8. Effect of different probe sizes on calculated ESA in lysozyme. (◯) K116, (◯) K97, (◯) K33, (◯) K1, (◯) K13, (◯) K96.

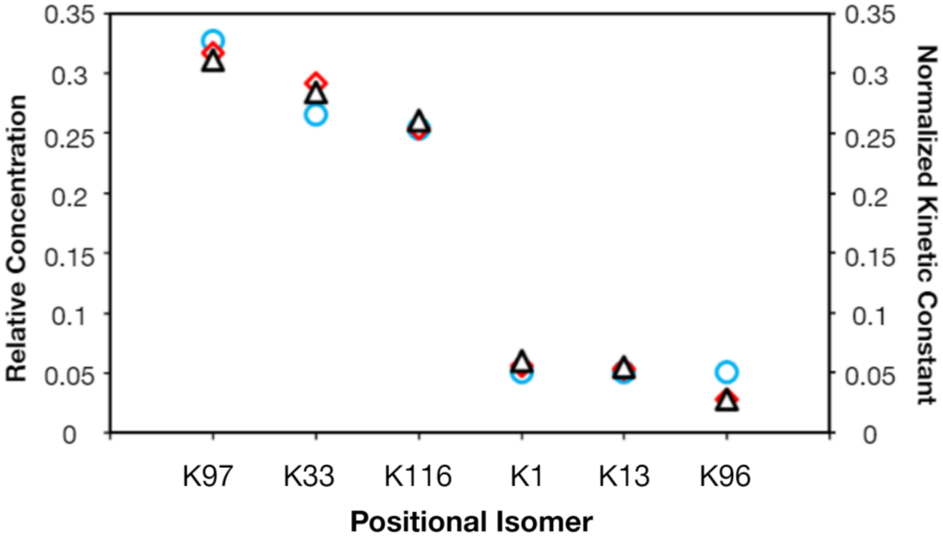

Figure S9. The concentration ratios of experimentally observed PEG positional isomers at different stoichiometry correspond well to the ratio of intrinsic rate constants of each residue, showing suitability in using intrinsic rate constant as a surrogate for predicting stochastic reaction sites. (○) Relative concentration of positional isomers with mPEG-linker to protein ratio 0.5:1, (◇) Relative concentration of positional isomers with mPEG-linker to protein ratio 1.5:1, (△) Normalized intrinsic kinetic constant for positional isomers. Literature data adapted from Pfister and co-workers. [1]

Table S1. Reactive site comparison with experimental results for tetrameric phenylalanine ammonia lyase (rAV-PAL) using a 20kDa N-hydroxysuccinimide PEG.[3]

Residue	Experimental	Linear Model 1		Machine Learned Model	
		Modified	Reactivity Rate ($M^{-1}.min^{-1}$)	Modified	Reactivity Rate ($M^{-1}.min^{-1}$)
K32	× (40%)	×	8.77	×	20.78
K109				×	6.45
K115	× (20%)	×	7.43	×	13.88
K145	× (50%)	×	5.20	×	7.06
K189					
K195	× (100%)	×	16.98	×	17.53
K216					
K272					
K301	× (40%)	×	11.60	×	20.46
K335	× (20%)	×	8.32	×	4.96
K384		×	7.24	×	12.08
K413	× (90%)	×	11.87	×	20.51
K419	× (20%)				
K493	× (100%)	×	10.97	×	20.76
K494	× (100%)	×	9.06	×	19.92
K522	× (100%)	×	15.54	×	6.65

Table S2. Reactive site comparison with experimental results for interferon-a 2a with an amine-reactive branched 40kDa PEG.[4] Due to a constraint (<24 Å) in probe size calculated with UCSF Chimera, a probe size equivalent to 20 kDa PEG was used.

Residue	Experimental	Linear Model 1		Machine Learned Model	
		Modified	Reactivity Rate ($M^{-1}.min^{-1}$)	Modified	Reactivity Rate ($M^{-1}.min^{-1}$)
C1		×	5.01		
K23		×	7.85	×	14.80
K31	×	×	33.63	×	20.89
K49	×	×	16.51	×	19.75
K70	×	×	32.73	×	16.35
K83	×	×	8.64	×	14.39
K112	×				
K121	×	×	10.03	×	19.25
K131	×	×	9.57	×	20.36
K133					
K134	×	×	33.49	×	16.35
K164	×	×	12.42	×	20.89

Normalisation of protein concentration during simulated PEGylation reaction (Figure 5)

Considering as an example a 5 kDa PEGylation reaction:

Relative concentrations were calculated by normalizing against the initial mass concentration of native protein. Thus, at t = 0, the native protein would always start at 1. If upon PEGylation, 2 moles of native protein were converted into 2 moles of a 5 kDa PEG-protein conjugate, then the relative concentration would change from

$$(2 \times Mw) \text{ to } [2 \times (Mw + 5\,000 \text{ Da})].$$

After normalisation, the relative concentration would increase to:

$$[(Mw + 5\,000 \text{ Da}) / Mw], \text{ which is greater than 1.}$$

Calculation of PEG conformation on lysozyme [5]

Lysozyme hydrodynamic radius[6] (r) = 2 nm

Surface area = $4\pi r^2$=50.24 nm^2

For tetra-grafted PEG, surface area per PEG = A = 50.24 nm^2 / 4 = 12.56 nm^2

Assuming the curvature is negligible, radius covered by each PEG chain = $\sqrt{A/\pi}$ = 2 nm

Distance between grafts = 2r = 4 nm

PEG monomers (N) = 110 and 220 for 5 and kDa PEG, respectively

R_f (5kDa) = $\alpha N^{3/5}$ = 0.35 nm x $110^{3/5}$ = 5.87 nm

R_f (10kDa) = $\alpha N^{3/5}$ = 0.35 nm x $220^{3/5}$ = 8.90 nm

R_f/D (5kDa) = 5.87 nm / 4 nm = 1.47

R_f/D (10kDa) = 8.90 nm / 4 nm = 2.23

REFERENCES

(1) Pfister, D.; Bourgeaux, E.; Morbidelli, M. Kinetic Modeling of Protein PEGylation. *Chem. Eng. Sci.* **2015**, *137*, 816–827.

(2) Munasinghe, A.; Baker, S. L.; Lin, P.; Russell, A. J.; Colina, C. M. Structure–Function–Dynamics of α-Chymotrypsin Based Conjugates as a Function of Polymer Charge. *Soft Matter* **2020**, *16* (2), 456–465.

(3) Bell, S. M.; Wendt, D. J.; Zhang, Y.; Taylor, T. W.; Long, S.; Tsuruda, L.; Zhao, B.; Laipis, P.; Fitzpatrick, P. A. Formulation and PEGylation Optimization of the Therapeutic PEGylated Phenylalanine Ammonia Lyase for the Treatment of Phenylketonuria. *PLOS ONE* **2017**, *12* (3), e0173269.

(4) Foser, S.; Schacher, A.; Weyer, K. A.; Brugger, D.; Dietel, E.; Marti, S.; Schreitmüller, T. Isolation, Structural Characterization, and Antiviral Activity of Positional Isomers of Monopegylated Interferon α-2a (PEGASYS). *Protein Expr. Purif.* **2003**, *30* (1), 78–87.

(5) Li, M.; Jiang, S.; Simon, J.; Paßlick, D.; Frey, M.-L.; Wagner, M.; Mailänder, V.; Crespy, D.; Landfester, K. Brush Conformation of Polyethylene Glycol Determines the Stealth Effect of Nanocarriers in the Low Protein Adsorption Regime. *Nano Lett.* **2021**, *21* (4), 1591–1598.

(6) Falke, S.; Dierks, K.; Blanchet, C.; Graewert, M.; Cipriani, F.; Meijers, R.; Svergun, D.; Betzel, C. Multi-Channel in Situ Dynamic Light Scattering Instrumentation Enhancing Biological Small-Angle X-Ray Scattering Experiments at the PETRA III Beamline P12. *J. Synchrotron Radiat.* **2018**, *25* (2), 361–372.
